# Early Postoperative Exposure to High-Fat Diet Does Not Increase Long-Term Weight Loss or Fat Avoidance After Roux-en-Y Gastric Bypass in Rats

**DOI:** 10.3389/fnut.2022.834854

**Published:** 2022-04-13

**Authors:** Aiman Ismaeil, Daniel Gero, Christina N. Boyle, Daniela Alceste, Osama Taha, Alan C. Spector, Thomas A. Lutz, Marco Bueter

**Affiliations:** ^1^Department of Surgery and Transplantation, University Hospital Zurich, Zurich, Switzerland; ^2^Department of General Surgery, Aswan University Hospital, Aswan, Egypt; ^3^Institute of Veterinary Physiology, University of Zurich, Zurich, Switzerland; ^4^Department of Plastic Surgery, Assiut University Hospital, Asyut, Egypt; ^5^Program in Neuroscience, Department of Psychology, Florida State University, Tallahassee, FL, United States

**Keywords:** bariatric (weight-loss) surgery, obesity, ingestive behavior, macronutrient intake, taste preference, conditioned taste avoidance, Roux-en-Y gastric bypass (RYGB), nutritional intervention

## Abstract

**Background:**

Bariatric surgery alters food preferences in rats and reportedly decreases desire to consume high-fat high-sugar food in humans. The aim of this study was to investigate whether early post-operative exposure to high-fat food could increase body weight loss after Roux-en-Y gastric bypass (RYGB) by triggering fat avoidance.

**Methods:**

Male Wistar rats underwent either RYGB (*n* = 15) or sham-operations (*n* = 16). Preoperatively a standardized 4-choice cafeteria diet [dietary options: low-fat/low-sugar (LFLS), low-fat/high-sugar (LFHS), high-fat/low-sugar (HFLS), high-fat/high-sugar (HFHS)] was offered. First, each option was available for 4 days, thereafter rats were offered the 4 options simultaneously for 3 days preoperatively. Post-surgery, 8 rats in the RYGB- and 8 in the sham-group were exposed to a high-fat content diet (Oatmeal + 30% lard, OM+L) for 10 days, while 7 RYGB rats and 8 sham-rats received OM alone. From the 11th postoperative day, the 4-choice cafeteria diet was reintroduced for 55-days. The intake of all available food items, macronutrients and body weight changes were monitored over 8 weeks. Main outcomes were long-term body-weight and daily change in relative caloric intake during the postoperative cafeteria period compared to the preoperative cafeteria.

**Results:**

During the first 12 days of postoperative cafeteria access, RYGB-rats exposed to OM+L had a higher mean caloric intake per day than RYGB rats exposed to OM alone (Δ10 kCal, *P*_adj_ = 0.004), but this difference between the RYGB groups disappeared thereafter. Consequently, in the last 33 days of the postoperative cafeteria diet, the mean body weight of the RYGB+OM+L group was higher compared to RYGB+OM (Δ51 g, *P*_adj_ < 0.001). RYGB rats, independently from the nutritional intervention, presented a progressive decrease in daily consumption of calories from fat and increased their daily energy intake mainly from non-sugar carbohydrates. No such differences were detected in sham-operated controls exposed to low- or high fat postoperative interventions.

**Conclusion:**

A progressive decrease in daily fat intake over time was observed after RYGB, independently from the nutritional intervention. This finding confirms that macronutrient preferences undergo progressive changes over time after RYGB and supports the role of ingestive adaptation and learning. Early postoperative exposure to high-fat food failed to accentuate fat avoidance and did not lead to superior weight loss in the long-term.

## Introduction

The easy availability of foods rich in sugar and fat is considered a significant contributor to the current obesity epidemic. Bariatric surgery (BS) (1) and especially Roux-en-Y gastric bypass (RYGB) effectively reduces food intake in patients with obesity (2–4), reaching on average a 40–50% decrease in energy intake 6 months postoperatively (5, 6). There are parallel physiological mechanisms responsible for postbariatric weight loss. These include increased postprandial gut hormone response (7–10) and intestinal vagal nerve signals that project to higher brain systems, leading to early satiation, and potentially altering the palatability of diets with high energy content, but whether this occurs is controversial (3, 9, 11–17). Despite the standardized surgical technique, the achieved weight loss shows wide inter-individual variations, suggesting that factors beyond the re-arranged gastro-intestinal anatomy heavily influence metabolic outcomes (18, 19).

There is a prevailing view that patients change food preferences after RYGB, and especially decrease consumption of high-fat and sweet items (20–28). However, this literature is dominated by verbal report of food intake. Indeed, recent studies that have applied more *direct* measures of food intake and choice have found little to any change in food selection or relative macronutrient energy intake (3, 29). On the other hand, in an *ad libitum* buffet meal test setting, administered preoperatively and at 6-month after BS, favorable changes in food preferences can be predicted with the use of a combination of factors, including genetic, psychologic and social patient characteristics (30). Beyond changes in food preferences, RYGB results also in lower average meal size. Our research group demonstrated that 1-year after RYGB, patients decreased the size of a liquid meal by 50% compared to preoperative baseline and needed 20% shorter time to reach satiation (6).

The rodent RYGB model is very effective for studying postbariatric ingestive behavior and to experimentally assess the effect of standardized nutritional interventions on weight loss (15, 31–33). Rodents after RYGB surgery do lose weight, consume fewer overall calories, and exhibit blunted preferences for food and fluids high in fat or sugar (14, 33–39). There are several standard behavioral tests that have been applied in rodent models of BS to assess changes in ingestive behavior. In an *ad libitum* setting after RYGB, rats show a decrease in intake of calorically dense solutions relative to that of water compared with controls (34–36, 40). Moreover, when a vegetable drink was used as an alternative to water and pitted with either fatty or sweet solutions, RYGB rats shifted their preferences to the vegetable drink (41). Changes in diet choices in rats after RYGB have been shown to be progressive, suggesting that learning from the consequences of intake over time affects ingestive behavior (39, 42, 43). Le Roux et al. observed that a conditioned taste avoidance was inducible in RYGB rats with oral gavage of corn oil, and therefore postulated that learned decreases in relative fat intake may contribute to long-term maintained weight loss after gastric bypass (36).

Against this theoretical framework, we aimed to investigate whether an early postoperative nutritional intervention consisting of an exposure to high-fat diet had the potential to promote and/or accelerate changes in food selection away from foods high in fat and ultimately would lead to a greater body weight loss in rats after RYGB. If our hypothesis were true, clinical bariatric practice could be improved by providing a nutritional therapy functioning as an adjuvant treatment to BS and helping patients in achieving superior weight loss.

## Materials and Methods

### Animals

Adult male Wistar rats (*n* = 43; RYGB+Oatmeal: 7, RYGB+Oatmeal+Lard: 8, Sham+Oatmeal: 8, Sham+Oatmeal+Lard: 8, prematurely euthanized and excluded from the study: 12) were used in the experiment. Rats were raised on standard laboratory chow (5001 Rodent Diet, Lab Diet, St. Louis, MO, United States) and had a mean baseline body weight of 319 ± 6 g. Rats were individually housed in single metal housing with a grid floor lined with white tissue to minimize food spillage and a sleeping tube. Room temperature was kept at 21 ± 2°C with humidity-controlled vivarium with 12-h automated light cycle. Wood pieces and cartoon sheets were placed for environmental enrichment. All experiments were performed under a license issued by the Veterinary Office of the Canton of Zurich, Switzerland ZH096/17.

### Diets

The food hoppers were placed in the cages. Four types of commercially available pelleted diets were used in the pre- and postoperative cafeteria diet phases: LFLS (low-fat low-sugar), LFHS (low-fat high-sugar), HFLS (high-fat low-sugar), and HFHS (high-fat high-sugar) (Supplementary Figure 1). For the postoperative nutritional intervention (postoperative days 6–16) rats were presented with either oatmeal (OM) or oatmeal plus lard (OM+L). All diets used in this study were manufactured by ssniff Spezialdiäten GmbH (Soest, Germany) with the respective macronutrient compositions shown in Table 1. Intake from all available food items and body weight changes were daily monitored throughout a total period of 8 weeks. Food spillage was collected and food intake was corrected accordingly.

**TABLE 1:** Energy density (kCal/g) and macronutrient composition (kCal/g) of the diets used in the study.

Diets	Caloric density kCal/g	Protein kCal/g (%kcal)	Fat kCal/g (%kcal)	Carbohydrate kCal/g (%kcal)	Sucrose kCal/g (%kcal) (included in carbohydrates)
Low-fat low-sugar (LFLS)	3.91	0.636 (16.3)	0.549 (14.0)	2.692 (68.8)	0.052 (1.3)
Low-fat high-sugar (LFHS)	3.91	0.636 (16.3)	0.549 (14.0)	2.692 (68.8)	2.38 (60.9)
High-fat low-sugar (HFLS)	5.07	0.636 (12.5)	2.71 (53.5)	1.692 (33.4)	0.052 (1.0)
High-fat high-sugar (HFHS)	5.07	0. 636 (12.5)	2.71 (53.5)	1.692 (33.4)	1.396 (27.5)
Oatmeal (OM)	3.17	0.24 (13.9)	0.25 (14.7)	1.23 (71.37)	0.05 (2.75)
Oatmeal + 30% Lard (OM+L)	5.2	0.39 (9.2)	1.86 (43.4)	2 (47.32)	0.08 (1.82)

### Surgery

All surgical procedures were performed under general anesthesia by one surgeon (AI) with a standardized and previously described RYGB and sham surgery technique (44, 45). Rats undergoing a RYGB had a mean preoperative body weight of 409 ± 7 g, while rats of the sham group weighted 408 ± 12 g. Rats were shaved, disinfected and placed on a heating pad during surgery to avoid hypothermia. On postoperative days 1–3, rats received subcutaneous injections of Baytril^®^ (enrofloxacin, 0.4 mL/kg) and meloxicam (1 mg/kg) for antibiotic prophylaxis and analgesia. Health checks were done daily on all animals. Twelve animals died prematurely and were excluded from the study analyses: 8 due to intra-operative anesthesiology complications, 1 in the RYGB group was euthanized due to gastrointestinal leak, and 3 RYGB rats were euthanized according to animal welfare regulations for higher than expected postoperative weight loss.

### Procedures

**FIGURE 1:**
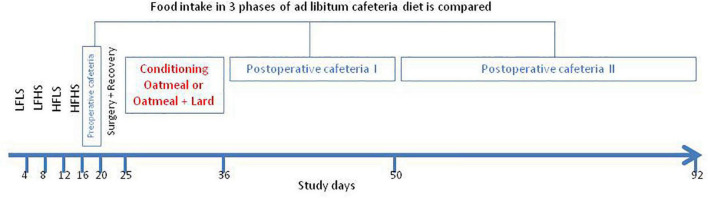
Schematic illustration of the study protocol. Diets: LFLS, low-fat low-sugar; LFHS, low-fat high-sugar; HFLS, high-fat low-sugar; and HFHS, high-fat high-sugar; Cafeteria diet: All four food items *ad libitum* available.

Upon arrival to our laboratory, before the start of the experiment, rats received normal chow during a 1-week acclimatization period. Then, starting at day 1, the four different diets (LFLS, LFHS, HFLS, and HFHS), as single source of food, were presented to the animals in a fixed order in 4-day intervals for 16 consecutive days (Figure 1). The goal of this phase was to familiarize the animals to each of the stimuli to reduce the likelihood of neophobia during the *ad libitum* cafeteria diet periods (46). Thereafter the 4 diets (preoperative cafeteria) were simultaneously available *ad libitum* for 3 days to prepare the animals for the post-operative phase with all four diets presented simultaneously (postoperative cafeteria diet during study days 37–92). Food was taken away on the day before surgery. Baseline absolute dietary intake and relative caloric intake from the three main macronutrients was computed based on mean values of the 3-day preoperative cafeteria period. To reduce location bias, food hoppers were rotated to each corner of the cage daily in a clockwise fashion. Fifteen rats received RYGB, while 16 underwent sham procedures. Postoperatively, all rats received a moist mesh chow diet for 5 days (recovery period) during which food intake was not monitored. Thereafter both RYGB and sham rats were randomly grouped to receive OM or OM+L for 10 days, as a single source of food. The postoperative cafeteria diet started immediately thereafter and lasted for 55 days, until study day 92. Intake from all diets and body weight were measured daily between 09.00 and 11.00 am throughout the entire study period. Water remained available *ad libitum* during the entire study.

### Data Analysis

The primary outcomes of the study consisted of the between group differences in body weight and in calorie intake (kCal) among the 4 treatment groups [surgery (RYGB or sham) × nutritional intervention (OM or OM+L)] during the 3 *ad libitum* cafeteria diet periods. The first period represented the 3-day pre-operative cafeteria phase (i.e., study days: 17–19). The 55-day long postoperative cafeteria phase was split *post hoc* to two phases (study days 37–48 phase I and 49–92 phase II). The cutpoint of division of the postoperative cafeteria diet into two phases was based on the stabilization of the body weight of the RYGB+OM+L group, which represents the turning point from the postoperative weight loss and compensatory weight regain phase into a metabolically stable phase of weight maintenance. To assess the progressivity in food preference changes, we used data visualization techniques showing food intake at each study days and statistical analyses comparing mean daily values during these three distinct cafeteria diet phases. Results are presented visually with group mean ± standard error and with individual values of each rat, as well as statistically using two-way ANOVA [independent variables = treatment group (Sham+OM, Sham+OM+L, RYGB+OM, and RYGB+OM+L) and cafeteria diet phases (preoperative, postoperative I and II)] with Bonferroni’s corrections for multiple comparisons. Data analysis was performed with the R software version 4.1.1 (R Foundation for Statistical Computing, Vienna, Austria).

## Results

**FIGURE 2:**
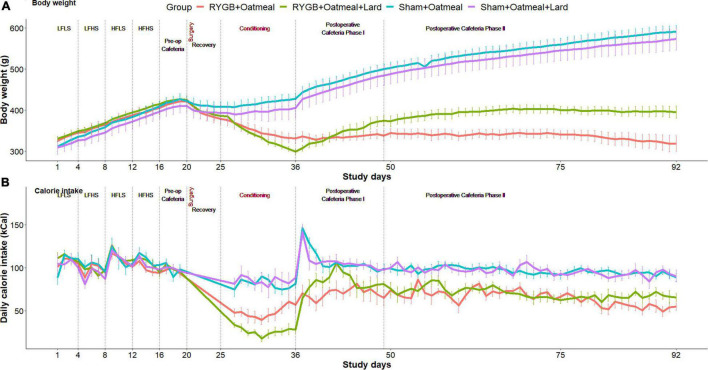
Body weight evolution **(A)** and daily calorie intake **(B)** in the four groups during the entire study. Study phases are separated with dotted lines.

Body weight changes and daily calorie intake during the different phases of the study are shown for each group in Figure 2. During the 10 days of postoperative exposure to OM or OM+L the caloric intake of sham rats did not depend on the nutritional intervention. In contrast, RYGB rats in the OM+L group consumed significantly (*P* < 0.001) less calories than RYGB rats in the OM group and compared to sham-operated rats.

**FIGURE 3:**
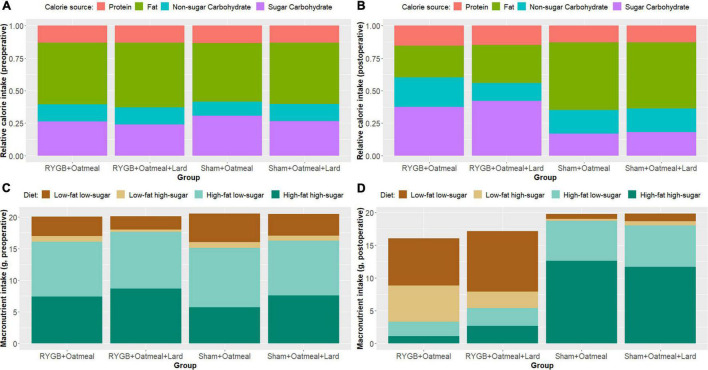
Overview of relative and absolute calorie intake from the three main macronutrients (fat, protein, and carbohydrates), and of food pellet diet choices in grams, representing main macronutrient source, during the 3-day preoperative cafeteria phase **(A,C)** and the 55-day postoperative cafeteria phase **(B,D)**.

Figures 3A,B show the relative mean caloric intake from the available diets in each group during the 3-day preoperative vs. the entire 55-day postoperative cafeteria phases. Preoperative food choices did not differ between the four groups; however, the postoperative food intake of sham rats was almost solely based on high-fat diet (HFHS+HFLS) (Figures 3C,D). On the contrary, postoperative diet for RYGB rats was mainly based on low-fat diet (LFLS+LFHS), with a higher daily consumption of HFHS food items in the RYGB+OM+L group. This led to a decreased relative intake of calories from fat after RYGB (mean change: −28.85%, *t* = 37.254, df = 1, *p*-value = 0.01708, 95% CI 19–38.7) when averaged over the entire postoperative cafeteria phase, while the proportion of intake from carbohydrates increased. In contrast, sham-operated rats changed their intake in an opposite way, by increasing the proportion of calories from fat and decreasing the proportion of ingested carbohydrates, independently from the nutritional intervention they received. The intrinsic discrepancy in relative caloric intake between the pre- vs. postoperative cafeteria diet phases was 0.0887 based on the Kullback–Leibler divergence function, a metric of differences between 2 probability distributions (47).

**FIGURE 4:**
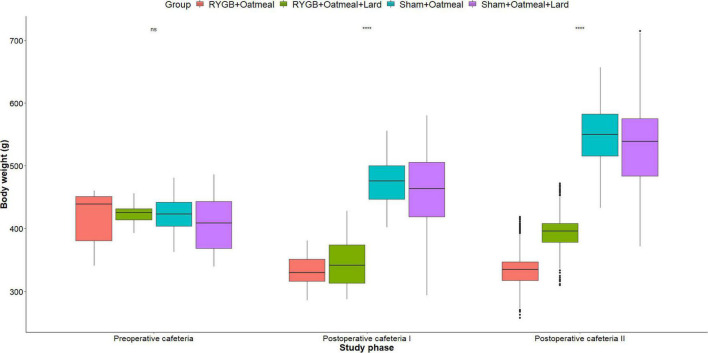
Mean body weight (g) during the three phases of the cafeteria diet in the four groups, with data presented as box-and-whisker plots where outliers are plotted as individual points beyond the whiskers on the box-plot. Statistics (two-way ANOVA with Bonferroni correction) were performed to investigate between group difference during these distinct study phases, significant differences are marked with ****; ns, non-significant.

**FIGURE 5:**
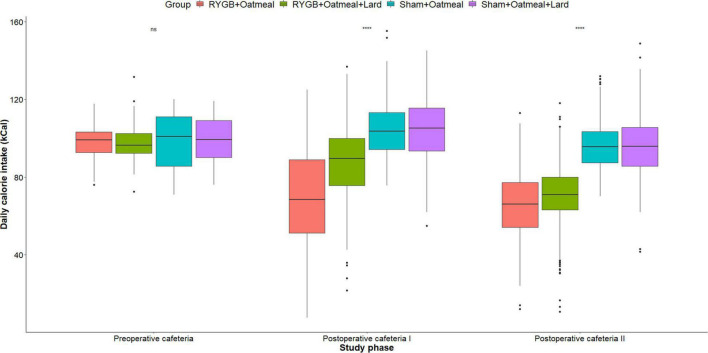
Mean daily total calorie (kCal) intake during the three phases of the cafeteria diet in the four groups. Statistics (two-way ANOVA with Bonferroni correction) were performed to investigate between group difference during these distinct study phases. ****, significant differences; ns, non-significant.

**FIGURE 6:**
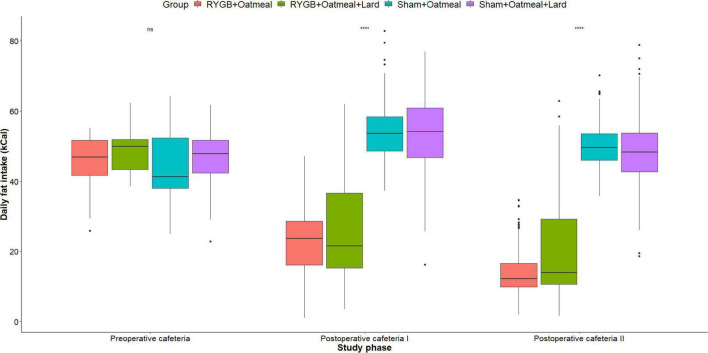
Mean daily calorie (kCal) intake from Fat during the three phases of the cafeteria diet in the four groups. Statistics (two-way ANOVA with Bonferroni correction) were performed to investigate between group difference during these distinct study phases. ****, significant differences; ns, non-significant.

Figures 4–6 and Tables 2–4 present the main outcomes of the study including comparisons of mean values between the groups during the 3 distinct cafeteria diet periods. During the postsurgical cafeteria phase (study days 37–92), sham rats steadily increased their body weight, while the body weight of RYGB rats was rather stable and below pre-operative baseline (Figures 2, 4). In contrast to our hypothesis, RYGB rats postoperatively exposed to OM+L had significantly less reduction in body weight by the end of the study and during the postoperative cafeteria phase II than those receiving OM alone. The total calorie consumption was mainly influenced by the surgical intervention, but not by the nutritional intervention (Figure 5). The *post hoc* test on total calorie intake revealed a non-significant difference between subgroups of sham rats, but identified a significant difference of +10 kCal/day in favor of RYGB+OM+L vs. RYGB+OM alone in postoperative cafeteria phase I (study days 37–48; *P* = 0.004) (Supplementary Figure 2). In the phase II of the postoperative cafeteria diet (study days 49–92), there was no significant difference in total caloric intake between the two RYGB groups. Among the three main macronutrients, the relative daily intake of calories from fat decreased the most after RYGB vs. sham, with a minor, but significant difference between the groups receiving OM or OM+L in the postoperative cafeteria phase II, with a more pronounced decrease in the RYGB+OM group (Figure 6). Changes in relative intake of calories from fat and carbohydrate to the total daily calorie intake, compared to the preoperative baseline is shown in the Supplementary Figure 3. Absolute changes in the consumption of the four diets (LFLS, LFHS, HFLS, and HFHS) are presented in Supplementary Figure 4, whereas the absolute and relative changes in caloric intake from the main macronutrient sources are presented in Supplementary Figures 2, 4. Statistical analyses of food intake during each sub-period of the study are shown in Supplementary Tables 1, 2.

**TABLE 2:** Comparison of mean body weight (g) during the three phases of the cafeteria diet in the four groups.

Cafeteria phase	Group 1	Group 2	Delta g (Group 1 – Group 2)	*P*. adj
Preoperative cafeteria	RYGB+Oatmeal	RYGB+Oatmeal+Lard	−0.47	1.00
Preoperative cafeteria	RYGB+Oatmeal	Sham+Oatmeal	−0.32	1.00
Preoperative cafeteria	RYGB+Oatmeal	Sham+Oatmeal+Lard	0.74	1.00
Preoperative cafeteria	RYGB+Oatmeal+Lard	Sham+Oatmeal	0.16	1.00
Preoperative cafeteria	RYGB+Oatmeal+Lard	Sham+Oatmeal+Lard	1.26	1.00
Preoperative cafeteria	Sham+Oatmeal	Sham+Oatmeal+Lard	1.09	1.00
Postoperative cafeteria I	RYGB+Oatmeal	RYGB+Oatmeal+Lard	−1.34	1.00
Postoperative cafeteria I	RYGB+Oatmeal	Sham+Oatmeal	−18.96	<0.001
Postoperative cafeteria I	RYGB+Oatmeal	Sham+Oatmeal+Lard	−16.72	<0.001
Postoperative cafeteria I	RYGB+Oatmeal+Lard	Sham+Oatmeal	−18.24	<0.001
Postoperative cafeteria I	RYGB+Oatmeal+Lard	Sham+Oatmeal+Lard	−15.93	<0.001
Postoperative cafeteria I	Sham+Oatmeal	Sham+Oatmeal+Lard	2.31	0.13
Postoperative cafeteria II	RYGB+Oatmeal	RYGB+Oatmeal+Lard	−15.80	<0.001
Postoperative cafeteria II	RYGB+Oatmeal	Sham+Oatmeal	−58.10	<0.001
Postoperative cafeteria II	RYGB+Oatmeal	Sham+Oatmeal+Lard	−53.70	<0.001
Postoperative cafeteria II	RYGB+Oatmeal+Lard	Sham+Oatmeal	−43.78	<0.001
Postoperative cafeteria II	RYGB+Oatmeal+Lard	Sham+Oatmeal+Lard	−39.23	<0.001
Postoperative cafeteria II	Sham+Oatmeal	Sham+Oatmeal+Lard	4.55	<0.001

*Statistics (two-way ANOVA with Bonferroni correction) were performed to investigate between group difference during these distinct study phases.*

**TABLE 3:** Comparison of mean daily total calorie (kCal) intake during the three phases of the cafeteria diet in the four groups.

Cafeteria phase	Group 1	Group 2	Delta (kCal Group 1 – Group 2)	*P*. adj
Preoperative cafeteria	RYGB+Oatmeal	RYGB+Oatmeal+Lard	−0.46	1.00
Preoperative cafeteria	RYGB+Oatmeal	Sham+Oatmeal	−0.19	1.00
Preoperative cafeteria	RYGB+Oatmeal	Sham+Oatmeal+Lard	−0.40	1.00
Preoperative cafeteria	RYGB+Oatmeal+Lard	Sham+Oatmeal	0.29	1.00
Preoperative cafeteria	RYGB+Oatmeal+Lard	Sham+Oatmeal+Lard	0.06	1.00
Preoperative cafeteria	Sham+Oatmeal	Sham+Oatmeal+Lard	−0.23	1.00
Postoperative cafeteria I	RYGB+Oatmeal	RYGB+Oatmeal+Lard	−5.68	<0.001
Postoperative cafeteria I	RYGB+Oatmeal	Sham+Oatmeal	−13.76	<0.001
Postoperative cafeteria I	RYGB+Oatmeal	Sham+Oatmeal+Lard	−13.41	<0.001
Postoperative cafeteria I	RYGB+Oatmeal+Lard	Sham+Oatmeal	−8.37	<0.001
Postoperative cafeteria I	RYGB+Oatmeal+Lard	Sham+Oatmeal+Lard	−8.00	<0.001
Postoperative cafeteria I	Sham+Oatmeal	Sham+Oatmeal+Lard	0.37	1.00
Postoperative cafeteria II	RYGB+Oatmeal	RYGB+Oatmeal+Lard	−4.12	<0.001
Postoperative cafeteria II	RYGB+Oatmeal	Sham+Oatmeal	−24.81	<0.001
Postoperative cafeteria II	RYGB+Oatmeal	Sham+Oatmeal+Lard	−24.49	<0.001
Postoperative cafeteria II	RYGB+Oatmeal+Lard	Sham+Oatmeal	−21.42	<0.001
Postoperative cafeteria II	RYGB+Oatmeal+Lard	Sham+Oatmeal+Lard	−21.08	<0.001
Postoperative cafeteria II	Sham+Oatmeal	Sham+Oatmeal+Lard	0.33	1.00

*Statistics (two-way ANOVA with Bonferroni correction) were performed to investigate between group difference during these distinct study phases.*

**TABLE 4:** Comparison of mean daily calorie (kCal) intake from Fat during the three phases of the cafeteria diet in the four groups.

Cafeteria phase	Group 1	Group 2	Delta kCal (Group 1 – Group 2)	*P*. adj
Preoperative cafeteria	RYGB+Oatmeal	RYGB+Oatmeal+Lard	−1.29	1.00
Preoperative cafeteria	RYGB+Oatmeal	Sham+Oatmeal	0.63	1.00
Preoperative cafeteria	RYGB+Oatmeal	Sham+Oatmeal+Lard	−0.24	1.00
Preoperative cafeteria	RYGB+Oatmeal+Lard	Sham+Oatmeal	2.00	0.28
Preoperative cafeteria	RYGB+Oatmeal+Lard	Sham+Oatmeal+Lard	1.09	1.00
Preoperative cafeteria	Sham+Oatmeal	Sham+Oatmeal+Lard	−0.91	1.00
Postoperative cafeteria I	RYGB+Oatmeal	RYGB+Oatmeal+Lard	−1.31	1.00
Postoperative cafeteria I	RYGB+Oatmeal	Sham+Oatmeal	−21.68	<0.001
Postoperative cafeteria I	RYGB+Oatmeal	Sham+Oatmeal+Lard	−21.28	<0.001
Postoperative cafeteria I	RYGB+Oatmeal+Lard	Sham+Oatmeal	−21.09	<0.001
Postoperative cafeteria I	RYGB+Oatmeal+Lard	Sham+Oatmeal+Lard	−20.67	<0.001
Postoperative cafeteria I	Sham+Oatmeal	Sham+Oatmeal+Lard	0.42	1.00
Postoperative cafeteria II	RYGB+Oatmeal	RYGB+Oatmeal+Lard	−8.62	<0.001
Postoperative cafeteria II	RYGB+Oatmeal	Sham+Oatmeal	−50.91	<0.001
Postoperative cafeteria II	RYGB+Oatmeal	Sham+Oatmeal+Lard	−48.81	<0.001
Postoperative cafeteria II	RYGB+Oatmeal+Lard	Sham+Oatmeal	−43.78	<0.001
Postoperative cafeteria II	RYGB+Oatmeal+Lard	Sham+Oatmeal+Lard	−41.60	<0.001
Postoperative cafeteria II	Sham+Oatmeal	Sham+Oatmeal+Lard	2.18	0.17

*Statistics (two-way ANOVA with Bonferroni correction) were performed to investigate between group difference during these distinct study phases.*

## Discussion

In this study, we applied an OM+L diet for 10 days, as an early postoperative nutritional intervention, with an attempt to increase avoidance of foods high in fat content and subsequently achieve greater weight loss after RYGB in rats. In contrast to our hypothesis, rats in the control group receiving an almost fat-free 10-day nutritional intervention after RYGB achieved superior long-term weight loss and lower daily energy intake when exposed to a four-choice cafeteria diet compared to rats undergoing RYGB along with the 10-day postoperative nutritional intervention including lard. Rats in the lard exposure group were unable to maintain the nadir of postoperative weight loss and consumed more food, mainly from the low-fat low-sugar diet option, representing an increased relative consumption of non-sugar carbohydrates compared to their own baseline levels and also in comparison to the other control groups.

The potential role of learning as a mechanism contributing to reduced fat and sugar intake after RYGB is further supported by studies in which a variety of human food items were presented in a “cafeteria”-style to rats before and after RYGB (38, 39). Under those conditions, RYGB rats took proportionally fewer calories from fat and more calories from non-sugar carbohydrates than sham rats. Our findings replicated, in principle, the observations from Mathes et al. (38) in which rats after RYGB progressively decreased the percentage of their daily calories taken from fat and at the same time, increased the percentage of calories taken from non-sugar carbohydrates from a cafeteria diet available over a period of 8 days after recovery from surgery. Importantly, our results extend the findings of the Mathes study by showing that such RYGB-induced changes in relative macronutrient intake are long-lasting, at least over the span of 66 postoperative days. Moreover, the foods employed in our study differed from those used in the Mathes et al. experiment, because they had less of a range of caloric densities and were all solid. Nevertheless, the outcomes in terms of changes in relative macronutrient intake after RYGB were similar between the two studies. The fact that rats after RYGB did not entirely stop consuming calories from the high-fat high-sugar diet option indicates that a conditioned taste aversion is not likely the driving force leading to postbariatric weight loss. Instead, we postulate that following RYGB, rats still find this food item quite palatable (6, 42), but due to the postingestive consequences (early and intense satiation signals), only a lower quantity is tolerated after RYGB, which would be in line with a conditioned taste avoidance (1, 17, 42, 43, 48, 49).

This assumption is also supported by data based on the analysis of the drinking microstructure in post-RYGB rats during short-term tests, where the animals decreased both their sucrose and Intralipid (fatty drink) intake across several days compared to sham-operated rats, and compared to their own preoperative baseline consumption. Despite the progressive decrease in fat and sugar intake observed in the RYGB group that occurred across sessions, early meal measures such as the first-minute lick rate or first burst size remained similar between rats that received RYGB or sham surgery (36). Because first-minute lick rate or first burst size are early meal measures supposedly reflecting palatability of the stimulus before the onset of significant postingestive consequences, it was hypothesized that the decreased overall intake in high fat high sugar stimuli represented a conditioned avoidance and not a decrease in palatability (36, 48, 50).

While learning is certainly implicated in the dietary choices made after RYGB, it was unexpected that an early and intense exposure of RYGB rats to fat increased overall energy intake, especially by a higher consumption of non-sugar carbohydrates from the cafeteria diet and led to a higher body weight at study end compared to the RYGB group not experiencing early exposure to lard. Of note, RYGB rats exposed to the OM+L diet consumed proportionally 6% fewer calories from fat compared to the RYGB+OM group initially. This suggests that rats of the OM+L group learned more effectively to avoid fat when compared to rats after RYGB who were exposed to OM alone. However, this effect was only of transient nature, as the extent of decrease in relative fat intake reversed between the two RYGB groups in the long-term in favor of the RYGB+OM group. In contrast, both sham groups increased their relative fat intake postoperatively; however, among sham-rats, exposure to OM+L led to a slightly lower increase in fat intake than OM alone. One explanation to these observations could be that RYGB rats undergoing the early high fat nutritional intervention may have immediately experienced taste avoidance, suggested by a lower caloric intake and a more pronounced weight loss during the 10 days of nutritional intervention compared to their counterparts receiving the low-fat intervention. Consequently, they may have compensated by overeating mainly carbohydrates during the postoperative *ad libitum* cafeteria period, which translated into weight regain and ultimately inferior long-term weight loss.

Our findings are also in line with Le Roux et al. (36), and Shin et al. (16), who observed reduced high-fat chow intake and increased low-fat chow intake 10 days after RYGB surgery relative to sham controls (36), which persisted also at 8 months after RYGB (16). Similar observations stem from research paradigms using non-caloric sweeteners in a two-bottle preference test in rats after RYGB, suggesting that a lower preference for fat and sucrose solutions relative to water is unlikely to be controlled solely by changes in taste function or unconditioned hedonic responsiveness. Rather these intake preferences appear to be driven by postingestive consequences and learning (34, 36, 41).

The collected evidence may form the basis for future investigations aiming to explore cellular, hormonal and neural consequences of bariatric surgery (43). It will be important to determine through direct measures whether the food preference shifts described here in rodents are also evident in humans. Recent studies suggest otherwise, but more work needs to be done (3, 13, 29, 30). Further understanding of the impact of bariatric operations on food preferences will certainly improve preoperative counseling and may help to optimize surgical outcomes (51, 52).

Strengths of the study include the use of commercially manufactured solid food pellets reducing measurement errors caused by food spillage or evaporation, which is frequently seen with the use of liquid diets. Another strength is related to the sequential introduction of each diet preoperatively, to reduce the effect of novelty during the pre- and postoperative cafeteria phases. The inclusion of sham groups in the design further supported the presence of dynamic changes in ingestive behavior due to RYGB and not to general anesthesia or surgery in general. Finally, the cafeteria diet test was conducted for 55 days postoperatively allowing for an assessment of the stability of the changes induced by RYGB. The main limitations of the study include the lack of monitoring of energy expenditure, of fecal energy loss and an absence of data related to the temporal structure of eating and drinking throughout the day and night (39, 53–55). Another limitation is related to the pre-exposure stimulus, which was a high-fat low-protein product. In humans almost every high-fat diet is also rich in protein (i.e., meat and dairy), therefore the extrapolation of our findings from rodents into human practice should be done with caution.

## Conclusion

A nutritional intervention in the form of oatmeal +30% lard early after RYGB failed to promote superior long-term weight loss outcomes in rats. Although the intervention led to a slightly more pronounced decrease in relative caloric intake from fat during the first 12 days of the postoperative cafeteria diet period, this effect reversed, and in the long term the RYGB group receiving the early postoperative exposure to lard had a higher daily relative fat intake compared to the respective controls. Nonetheless, a progressive decrease in daily fat intake over time was observed in all rats undergoing RYGB, while sham rats increased postoperatively their daily caloric intake from fat.

## Data Availability Statement

The raw data supporting the conclusions of this article will be made available by the authors, without undue reservation.

## Ethics Statement

The animal study was reviewed and approved by the Veterinary Office of the Canton of Zurich, Switzerland (ZH 096/17).

## Author Contributions

MB, TL, DG, AI, and AS: conception and design of the research. AI and CB: performing the experiment. AI: data collection. DG: data analysis and creation of figures. DG and AI: manuscript drafting. All authors contributed to the data interpretation, editing, revising, and approving the manuscript.

## Conflict of Interest

The authors declare that the research was conducted in the absence of any commercial or financial relationships that could be construed as a potential conflict of interest.

## Publisher’s Note

All claims expressed in this article are solely those of the authors and do not necessarily represent those of their affiliated organizations, or those of the publisher, the editors and the reviewers. Any product that may be evaluated in this article, or claim that may be made by its manufacturer, is not guaranteed or endorsed by the publisher.
