# Supplementary material for: Early Postoperative Exposure to High-Fat Diet Does Not Increase Long-Term Weight Loss or Fat Avoidance After Roux-en-Y Gastric Bypass in Rats
Source: Front Nutr. 2022 Apr 13;9:834854. doi: 10.3389/fnut.2022.834854 (PMC9044042; doi:10.3389/fnut.2022.834854)
Supplement: Supplementary file 1 [file Data_Sheet_1.PDF]

Supplemental Digital Content

Early postoperative exposure to high-fat diet does not increase long-term weight loss or fat avoidance after Roux-en-Y gastric bypass in rats. *Ismaeil, Gero et al.* Frontiers in Nutrition 2022

Supplementary Figures

Supplementary Figure 1. Colored pellet diets with varying fat and sucrose content used in the study during the pre- and postoperative cafeteria phase.

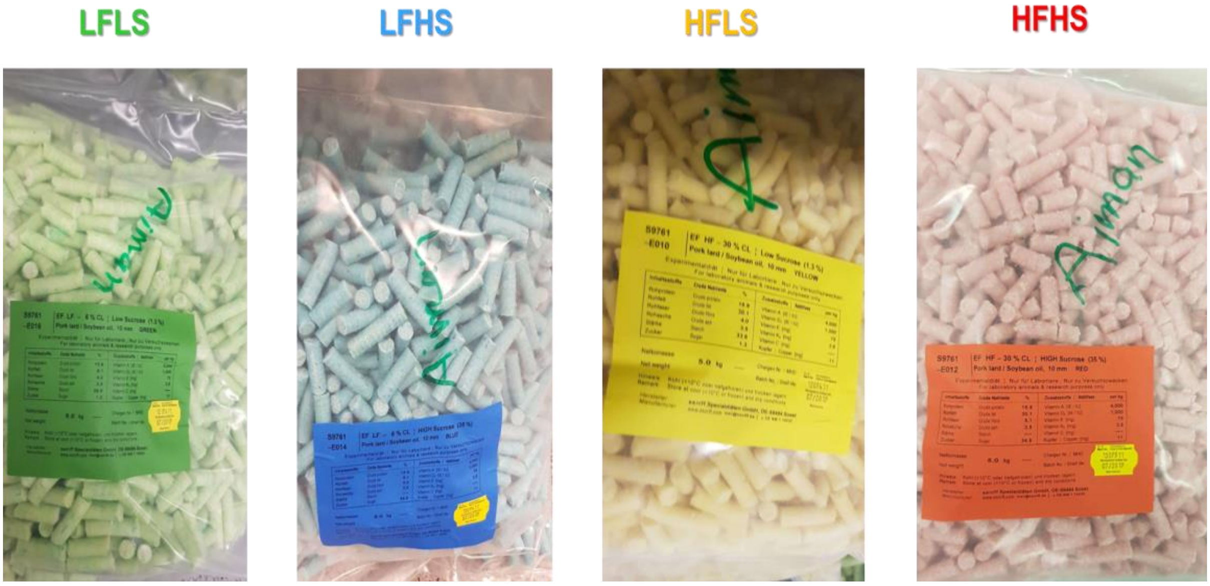

Supplementary Figure 2. Comparison of change/day in group means with 95% family-wise confidence level during the postoperative cafeteria period from the mean of the preoperative cafeteria period for primary outcomes. A. Body weight B. Total calories C. Fat calories D. Protein calories E. Carbohydrate calories F. Sugar calories.

A.

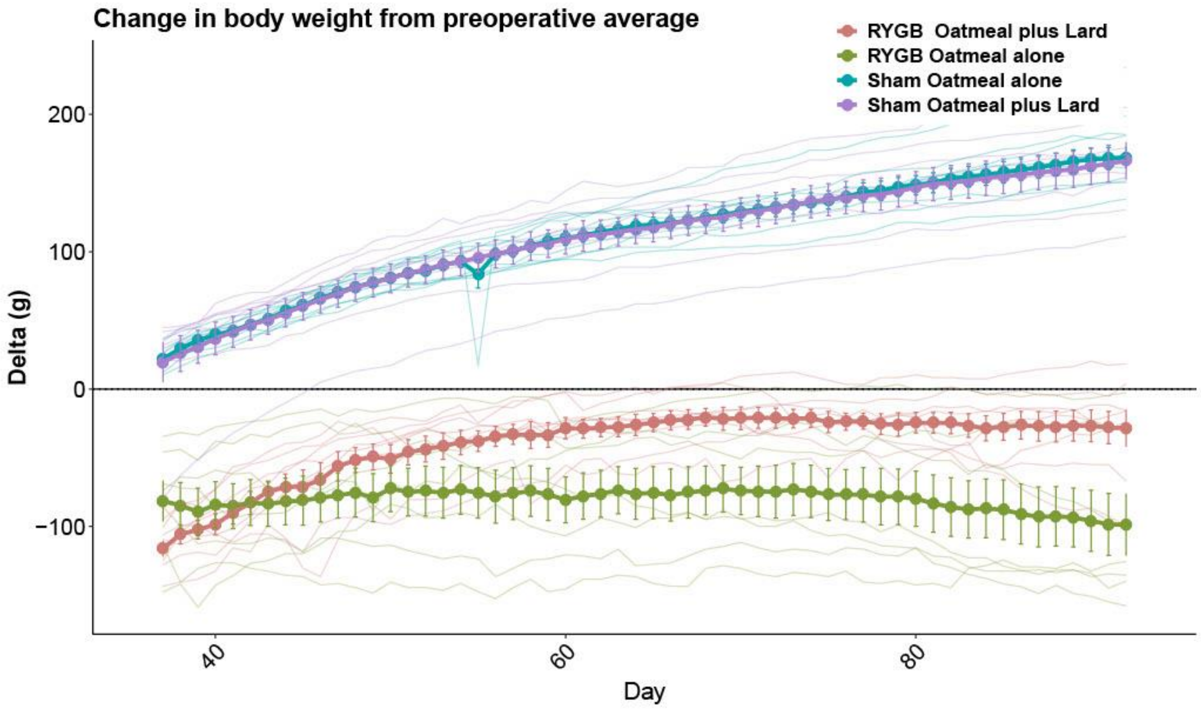

		Body weight (g)		
**Group A**	**Group B**	*Difference*	*95% CI*	*P adj*
**RYGB+Oatmeal**	**RYGB+Oatmeal Lard**	-40	-48 - -32.4	<0.0001
**Sham+Oatmeal**	**RYGB+Oatmeal Lard**	151.76	144.2 - 159.31	<0.0001
**Sham+Oatmeal Lard**	**RYGB+Oatmeal Lard**	150.4	142.9 - 157.9	<0.0001
**Sham+Oatmeal**	**RYGB+Oatmeal**	192	184.2 - 199.8	<0.0001
**Sham+Oatmeal Lard**	**RYGB+Oatmeal**	190.67	182.9 - 198.5	<0.0001
**Sham+Oatmeal Lard**	**Sham+Oatmeal**	-1.34	-8.9 - 6.2	0.96

B.

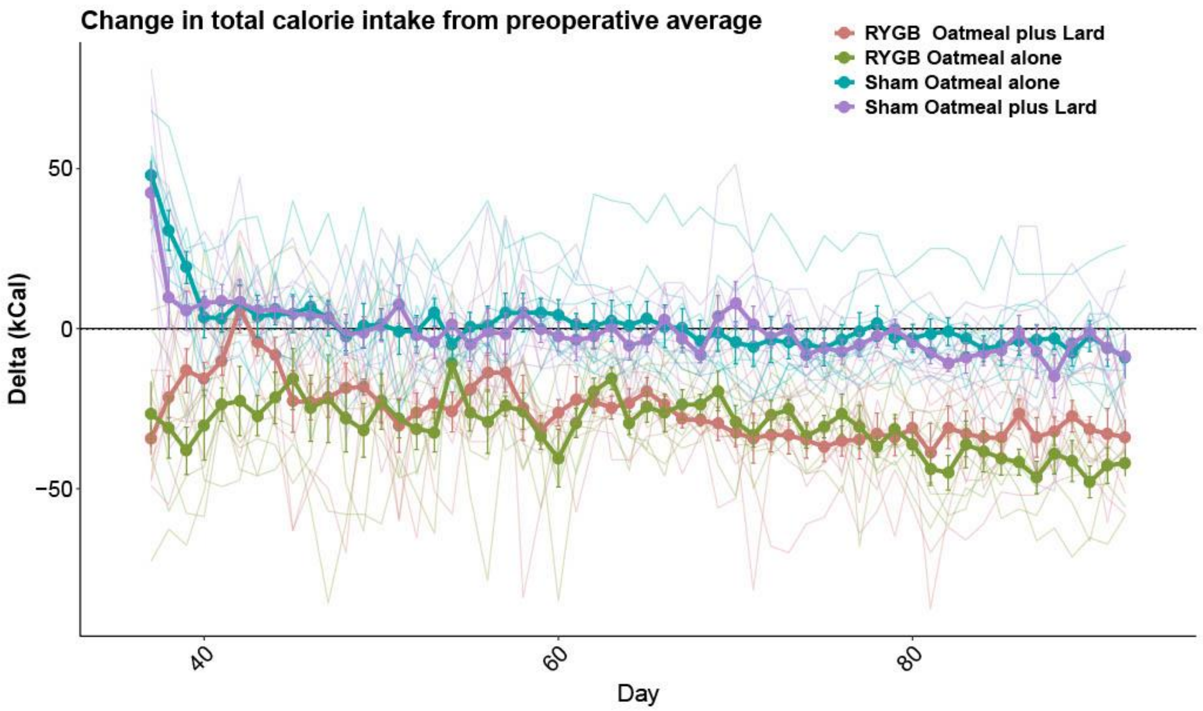

		Total calories (kCal)		
**Group A**	**Group B**	*Difference*	*95% CI*	*P adj*
**RYGB+Oatmeal**	**RYGB+Oatmeal Lard**	-4.6	-7.6 - -1.6	0.0006
**Sham+Oatmeal**	**RYGB+Oatmeal Lard**	27.11	24.2 - 30	<0.0001
**Sham+Oatmeal Lard**	**RYGB+Oatmeal Lard**	25.33	22.4 - 28.29	<0.0001
**Sham+Oatmeal**	**RYGB+Oatmeal**	31.71	28.7 - 34.74	<0.0001
**Sham+Oatmeal Lard**	**RYGB+Oatmeal**	29.93	26.9 - 32.97	<0.0001
**Sham+Oatmeal Lard**	**Sham+Oatmeal**	-1.77	-4.7 - 1.15	0.4

C.

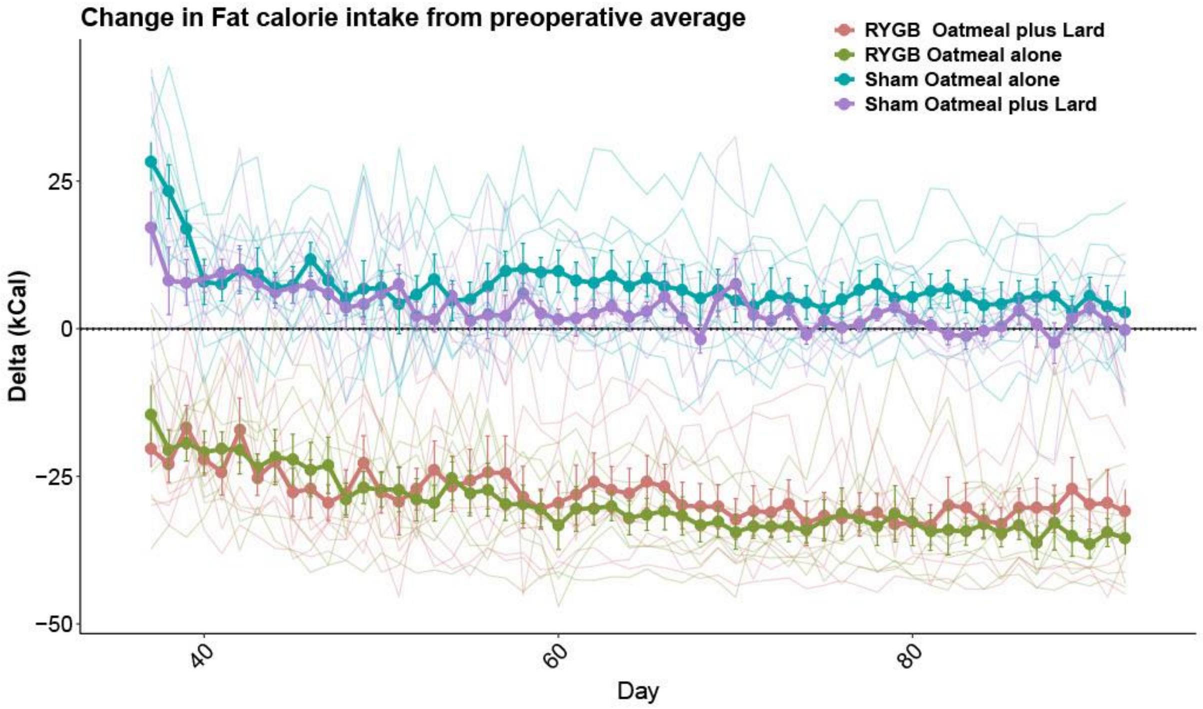

		Fat calories (kCal)		
**Group A**	**Group B**	*Difference*	*95% CI*	*P adj*
**RYGB+Oatmeal**	**RYGB+Oatmeal Lard**	-1.61	-3.38 - 0.15	0.088
**Sham+Oatmeal**	**RYGB+Oatmeal Lard**	35.42	33.7 -37.13	<0.0001
**Sham+Oatmeal Lard**	**RYGB+Oatmeal Lard**	31.57	29.86 - 33.28	<0.0001
**Sham+Oatmeal**	**RYGB+Oatmeal**	37.04	35.26 - 38.8	<0.0001
**Sham+Oatmeal Lard**	**RYGB+Oatmeal**	33.18	31.41 - 31.41	<0.0001
**Sham+Oatmeal Lard**	**Sham+Oatmeal**	-3.84	-5.55 - -2.14	<0.0001

D.

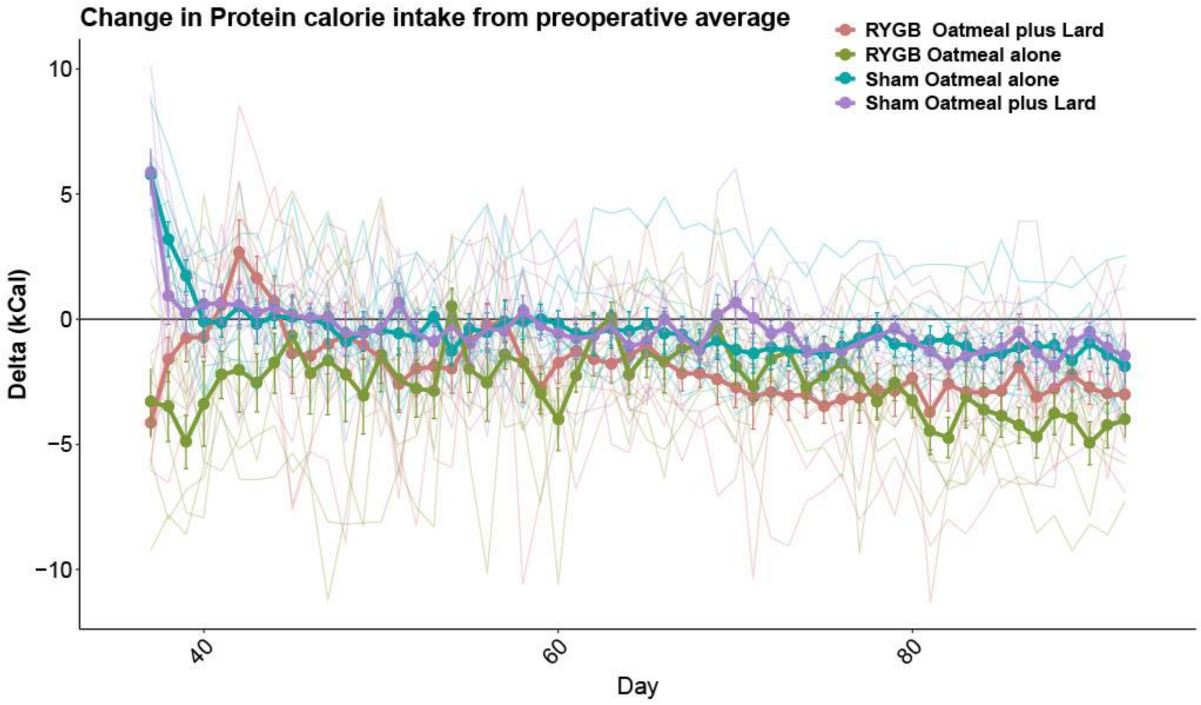

		Protein calories (kCal)		
**Group A**	**Group B**	*Difference*	*95% CI*	*P adj*
**RYGB+Oatmeal**	**RYGB+Oatmeal Lard**	-0.64	-1 - 0.1	0.0013
**Sham+Oatmeal**	**RYGB+Oatmeal Lard**	1.41	0.98 - 1.85	<0.0001
**Sham+Oatmeal Lard**	**RYGB+Oatmeal Lard**	1.5	1.07 - 1.94	<0.0001
**Sham+Oatmeal**	**RYGB+Oatmeal**	2.1	1.61 - 2.51	<0.0001
**Sham+Oatmeal Lard**	**RYGB+Oatmeal**	2.15	1.7 - 2.6	<0.0001
**Sham+Oatmeal Lard**	**Sham+Oatmeal**	0.09	-0.3 - 0.5	0.95

E.

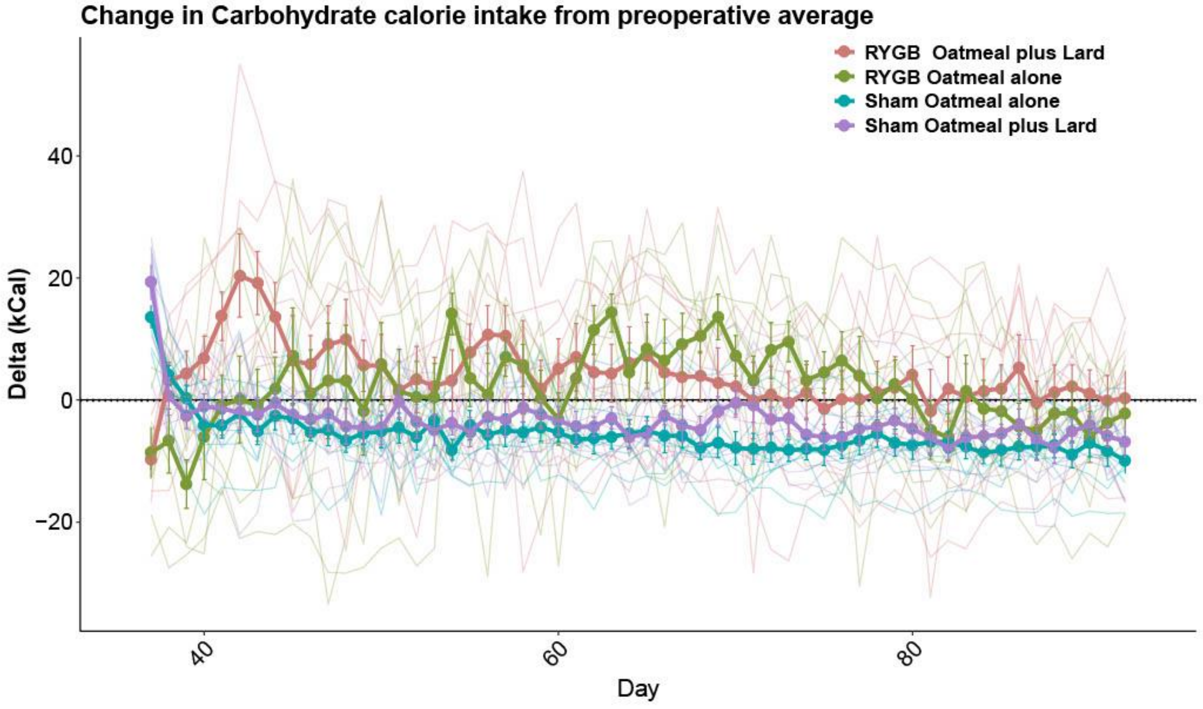

		Carbohydrates calories (kCal)		
**Group A**	**Group B**	*Difference*	*95% CI*	*P adj*
**RYGB+Oatmeal**	**RYGB+Oatmeal Lard**	0.01	-0.002 - 0.03	0.1
**Sham+Oatmeal**	**RYGB+Oatmeal Lard**	-0.26	-0.27 - -0.24	<0.0001
**Sham+Oatmeal Lard**	**RYGB+Oatmeal Lard**	-0.22	-0.24 - -0.2	<0.0001
**Sham+Oatmeal**	**RYGB+Oatmeal**	-0.27	- 0.24 - -0.2	<0.0001
**Sham+Oatmeal Lard**	**RYGB+Oatmeal**	-0.23	-0.25 - -0.22	<0.0001
**Sham+Oatmeal Lard**	**Sham+Oatmeal**	0.033	0.02 - 0.048	<0.0001

F.

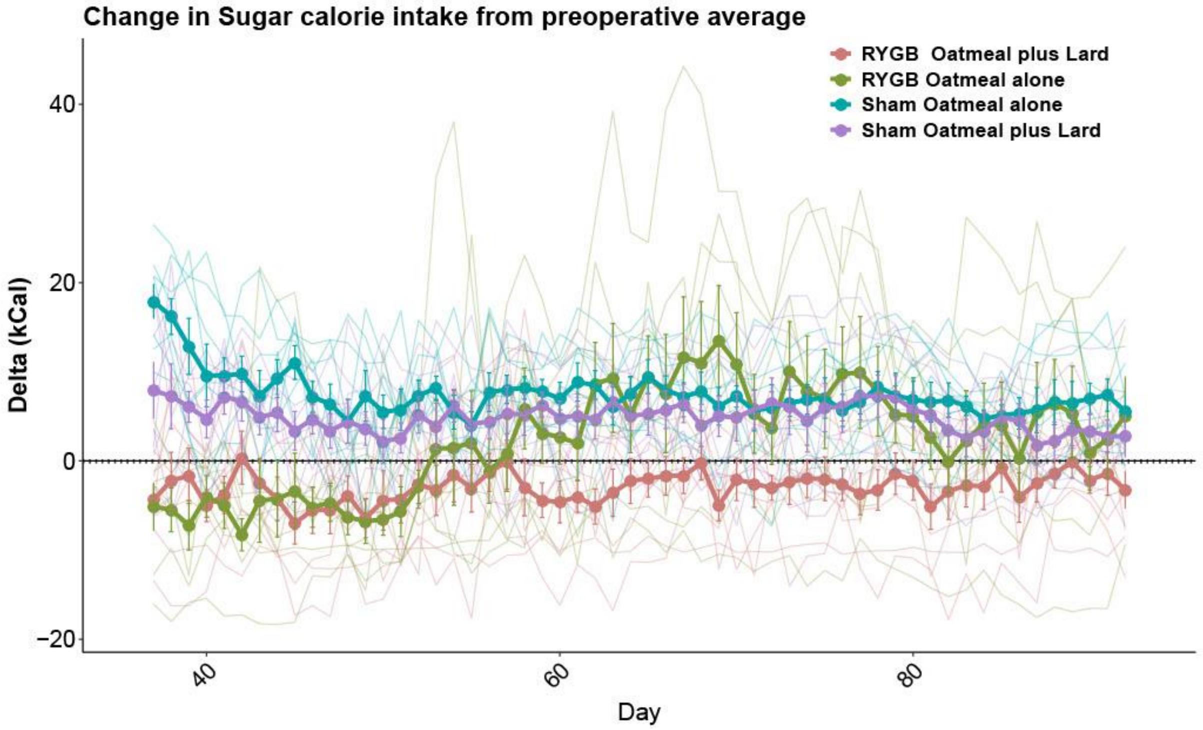

		Sugar calories (kCal)		
**Group A**	**Group B**	*Difference*	*95% CI*	*P adj*
**RYGB+Oatmeal**	**RYGB+Oatmeal Lard**	5.31	3.87 - 6.75	<0.0001
**Sham+Oatmeal**	**RYGB+Oatmeal Lard**	10.49	9 - 11.88	<0.0001
**Sham+Oatmeal Lard**	**RYGB+Oatmeal Lard**	7.89	6.5 - 9.3	<0.0001
**Sham+Oatmeal**	**RYGB+Oatmeal**	5.18	3.74 - 6.61	<0.0001
**Sham+Oatmeal Lard**	**RYGB+Oatmeal**	2.58	1.14 - 4.02	<0.0001
**Sham+Oatmeal Lard**	**Sham+Oatmeal**	-2.6	-3.98 - -1.2	<0.0001

Supplementary Figures 3. Change in relative intake of calories from A. Fat and B. Carbohydrate relative to the total daily calorie intake, compared to the preoperative baseline showed in the 4 groups.

A.

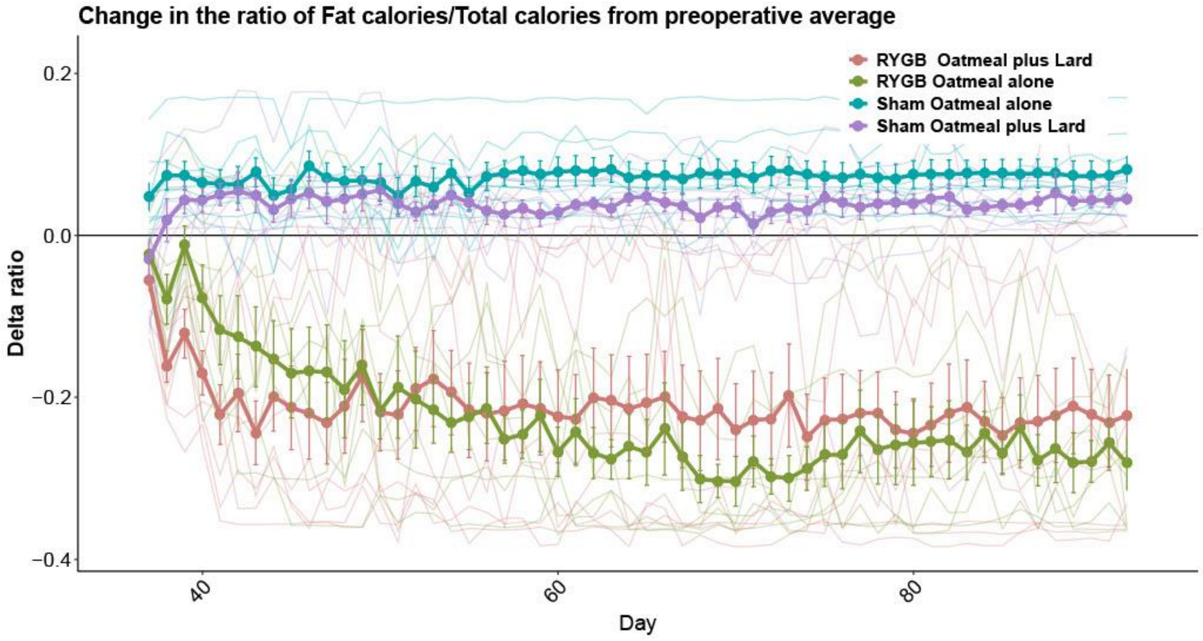

B.

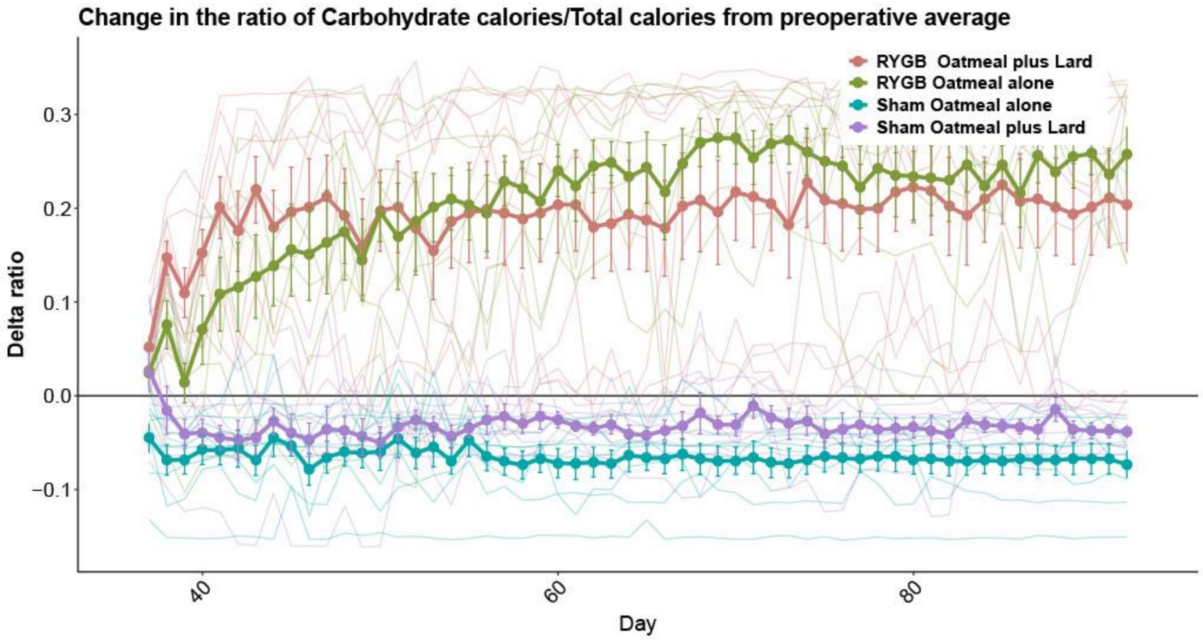

Supplementary Figures 4. Change/day in the intake of the 4 diets during the entire postoperative cafeteria period from the mean of the preoperative cafeteria period. A. Low fat low sugar B. Low fat high sugar C. High fat low sugar D. High fat high sugar

A.

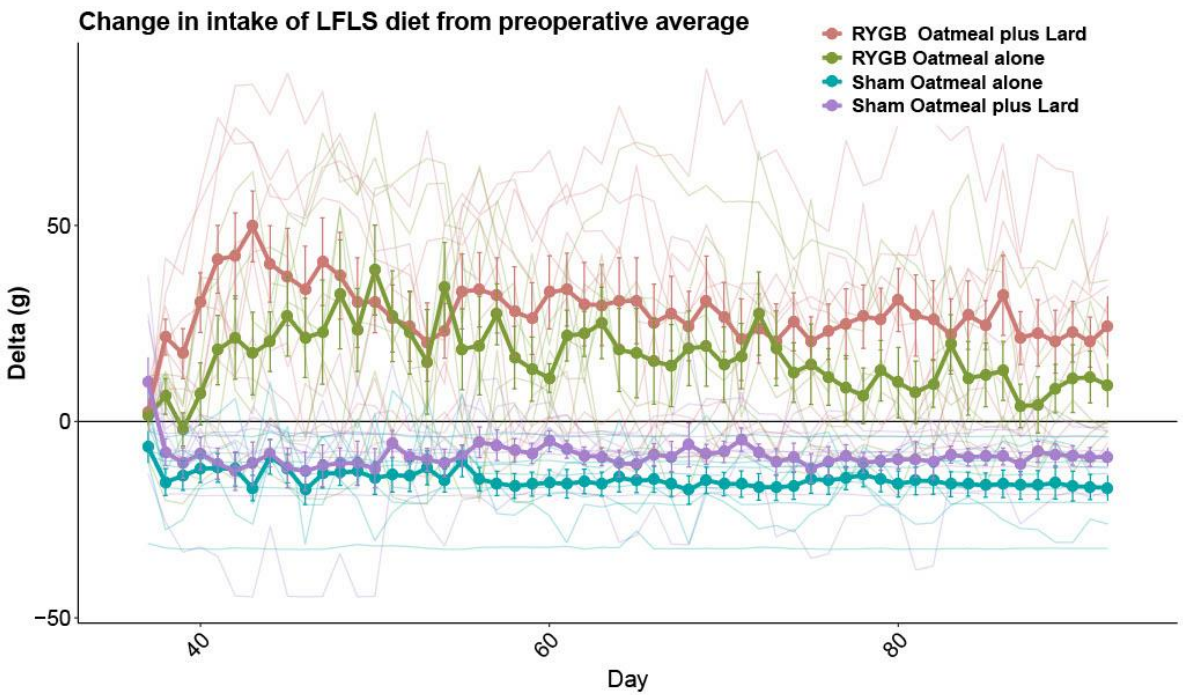

B.

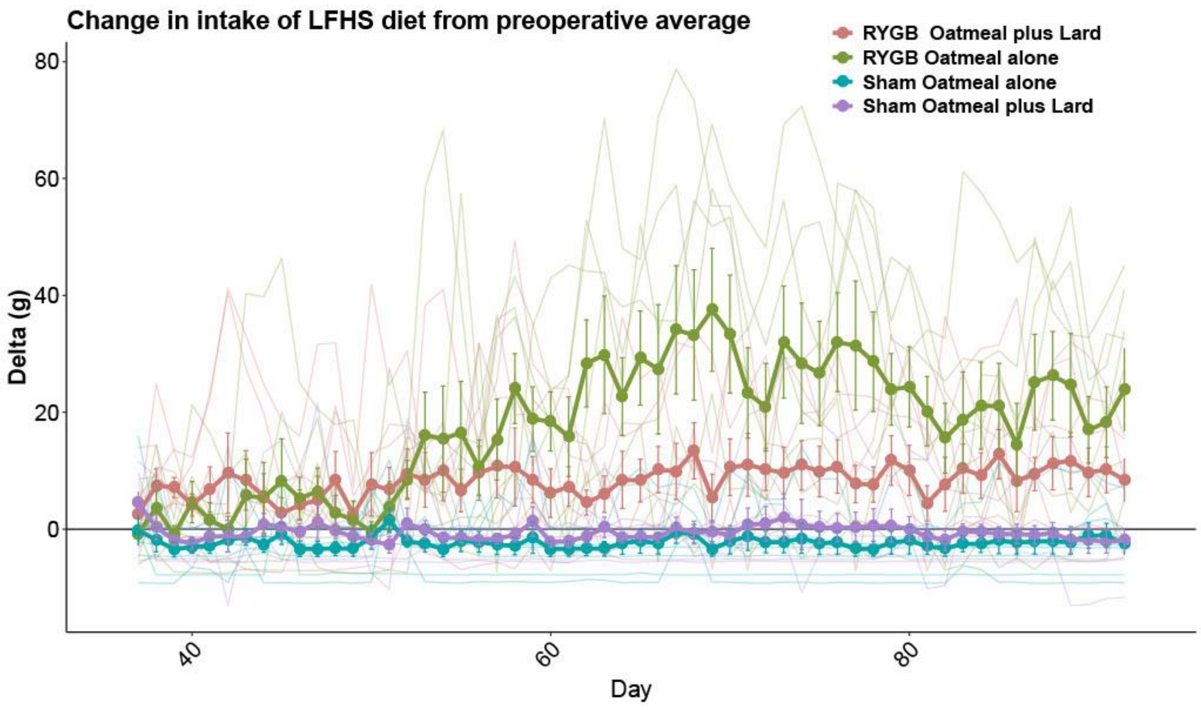

C.

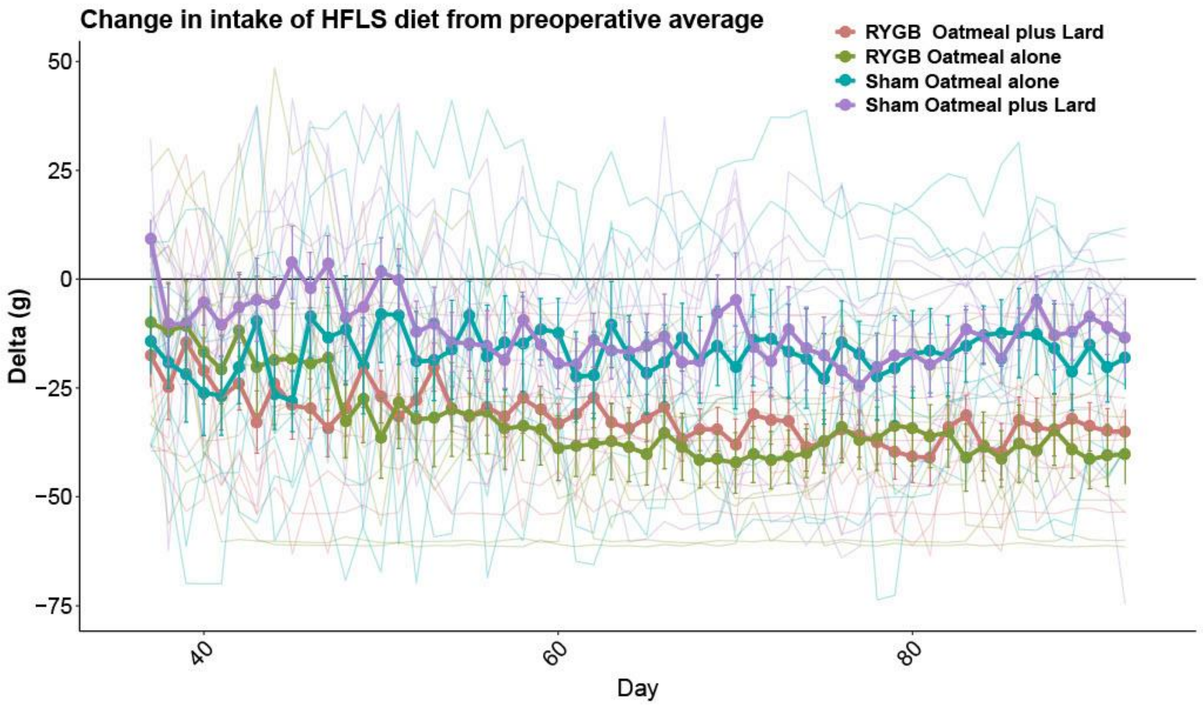
Change in intake of HFLS diet from preoperative average
RYGB Oatmeal plus Lard
RYGB Oatmeal alone
Sham Oatmeal alone
Sham Oatmeal plus Lard
Delta (g)
50
25
0
−25
−50
−75
40
60
80
Day

D.

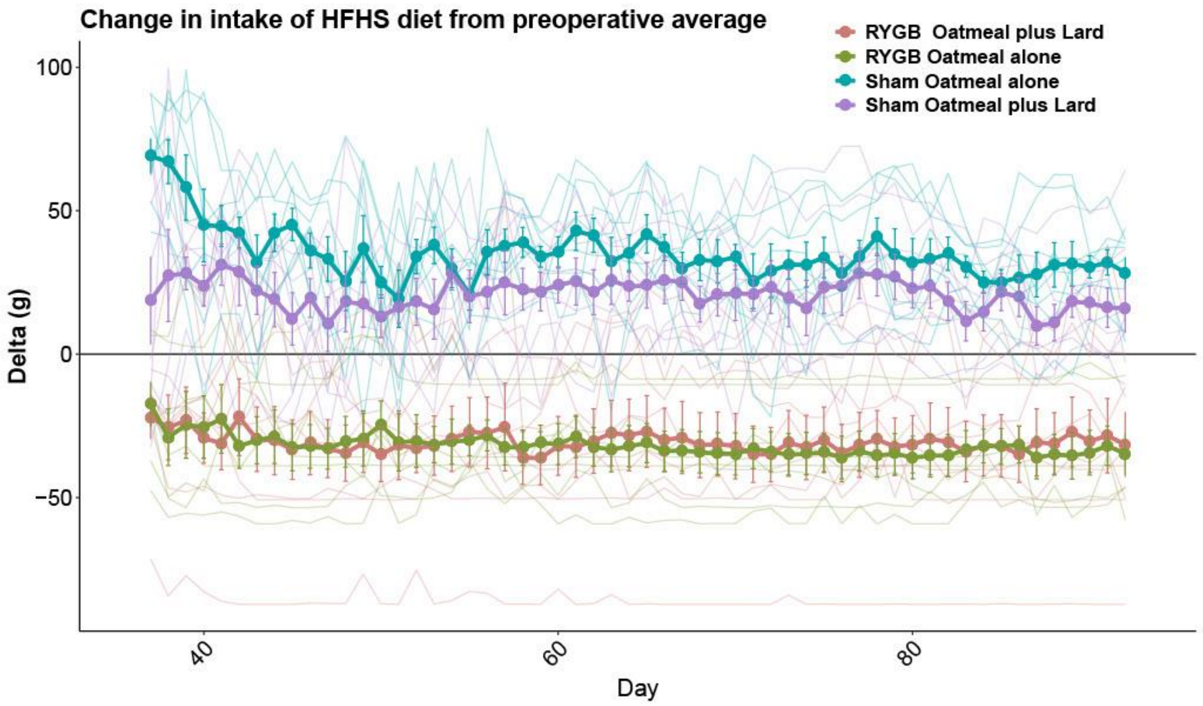
Change in intake of HFHS diet from preoperative average
RYGB Oatmeal plus Lard
RYGB Oatmeal alone
Sham Oatmeal alone
Sham Oatmeal plus Lard
Delta (g)
100
50
0
−50
40
60
80
Day

Supplementary Tables

Supplementary Table 1. Comparison of change/day in group means with 95% family-wise confidence level (one-way ANOVA with Tukey HSD) during the postoperative cafeteria period (Phase I: study days 37- 48 and Phase II: study days 49-92) from the mean of the preoperative cafeteria period for primary outcomes. A. Body weight B. Total caloric intake. *RYGB: Roux-en-Y gastric bypass, OM: Oatmeal, OM+L: Oatmeal+ Lard*

A. Postoperative cafeteria Phase I (study days 37 – 48)

		**Body weight (g)**		
**Group A**	**Group B**	*Difference*	*95% CI*	*P adj*
**RYGB+OM+L**	**RYGB+OM**	-0.01488	-12.36 – 12.32	1
**Sham+OM**	**RYGB+OM**	131.881	119.54 – 144.25	<0.001
**Sham+OM+L**	**RYGB+OM**	130.2872	117.94 – 142.63	<0.001
**Sham+OM**	**RYGB+OM+L**	131.8958	119.97 – 143.82	<0.001
**Sham+OM+L**	**RYGB+OM+L**	130.3021	118.37 – 142.22	<0.001
**Sham+OM+L**	**Sham+OM**	-1.59375	-13.52 – 10.33	0.98

Postoperative cafeteria Phase II (study days 49 – 92)

		**Body weight (g)**		
**Group A**	**Group B**	*Difference*	*95% CI*	*P adj*
**RYGB+OM+L**	**RYGB+OM**	51.23701	43.91 – 58.56	<0.001
**Sham+OM**	**RYGB+OM**	208.4198	201.09 – 215.74	<0.001
**Sham+OM+L**	**RYGB+OM**	207.1423	199.81 – 214-46	<0.001
**Sham+OM**	**RYGB+OM+L**	157.1828	150.1 – 164.26	<0.001
**Sham+OM+L**	**RYGB+OM+L**	155.9053	148.83 – 162.98	<0.001
**Sham+OM+L**	**Sham+OM**	-1.27746	-8.35 – 5.79	0.96

B. Postoperative cafeteria Phase I (study days 37 – 48)

		**Total calories (kCal)**		
**Group A**	**Group B**	*Difference*	*95% CI*	*P adj*
**RYGB+OM+L**	**RYGB+OM**	10.40476	2.47- 18.34	0.004
**Sham+OM**	**RYGB+OM**	37.09226	29.15 – 45.02	<0.001
**Sham+OM+L**	**RYGB+OM**	34.69643	26.75 – 42.63	<0.001
**Sham+OM**	**RYGB+OM+L**	26.6875	19.02 – 34.35	<0.001
**Sham+OM+L**	**RYGB+OM+L**	24.29167	16.62 – 31.95	<0.001
**Sham+OM+L**	**Sham+OM**	-2.39583	-10.064 – 5.27	0.85

Postoperative cafeteria Phase II (study days 49 – 92)

		**Total calories (kCal)**		
**Group A**	**Group B**	*Difference*	*95% CI*	*P adj*
**RYGB+OM+L**	**RYGB+OM**	3.01434	-0.019 – 6.04	0.052
**Sham+OM**	**RYGB+OM**	30.24256	27.20 – 33.27	<0.001
**Sham+OM+L**	**RYGB+OM**	28.6365	25.60 – 31.66	<0.001
**Sham+OM**	**RYGB+OM+L**	27.22822	24.30 – 30.16	<0.001
**Sham+OM+L**	**RYGB+OM+L**	25.62216	22.69 – 28.55	<0.001
**Sham+OM+L**	**Sham+OM**	-1.60606	-4.53 – 1.32	0.49

Supplementary Table 2. Change in relative intake of calories from A. Fat; B. Protein; C. Non-Sugar Carbohydrate and D. Sugar Carbohydrate relative to the total daily calorie intake, compared to the preoperative baseline showed in the 4 groups. Statistics (one-way ANOVA with Tukey HSD) were performed in the postoperative cafeteria period Phase I (study days 37- 48) Phase II (study days 49-92) to investigate between group difference in these distinct postoperative phases. *RYGB: Roux-en-Y gastric bypass, OM: Oatmeal, OM+L: Oatmeal+Lard*

A. Postoperative cafeteria Phase I (study days 37 – 48)

		**Ratio of calories from fat / total calories**		
**Group A**	**Group B**	*Difference*	*95% CI*	*P adj*
**RYGB+OM+L**	**RYGB+OM**	-0.06843	-0.10- -0.03	<0.001
**Sham+OM**	**RYGB+OM**	0.184629	0.147 – 0.221	<0.001
**Sham+OM+L**	**RYGB+OM**	0.155517	0.118 – 0.192	<0.001
**Sham+OM**	**RYGB+OM+L**	0.253056	0.217 – 0.288	<0.001
**Sham+OM+L**	**RYGB+OM+L**	0.223944	0.188 – 0.259	<0.001
**Sham+OM+L**	**Sham+OM**	-0.02911	-0.064 – 0.006	0.149

Postoperative cafeteria Phase II (study days 49 – 92)

		**Ratio of calories from fat / total calories**		
**Group A**	**Group B**	*Difference*	*95% CI*	*P adj*
**RYGB+OM+L**	**RYGB+OM**	0.037357	0.0182 – 0.056	<0.001
**Sham+OM**	**RYGB+OM**	0.329479	0.31 – 0.348	<0.001
**Sham+OM+L**	**RYGB+OM**	0.294013	0.27 – 0.313	<0.001
**Sham+OM**	**RYGB+OM+L**	0.292122	0.273 – 0.31	<0.001
**Sham+OM+L**	**RYGB+OM+L**	0.256657	0.238 – 0.275	<0.001
**Sham+OM+L**	**Sham+OM**	-0.03547	-0.054 - -0.017	<0.001

B. **Postoperative cafeteria Phase I (study days 37 – 48)**

		**Ratio of calories from protein / total calories**		
**Group A**	**Group B**	*Difference*	*95% CI*	*P adj*
**RYGB+OM+L**	**RYGB+OM**	0.006085	0.0026 – 0.009	<0.001
**Sham+OM**	**RYGB+OM**	-0.01835	-0.022 - -0.015	<0.001
**Sham+OM+L**	**RYGB+OM**	-0.01527	-0.019 - -0.012	<0.001
**Sham+OM**	**RYGB+OM+L**	-0.02444	-0.028 - -0.021	<0.001
**Sham+OM+L**	**RYGB+OM+L**	-0.02135	-0.025 - -0.02	<0.001
**Sham+OM+L**	**Sham+OM**	0.003088	0.00 – 0.006	0.086

Postoperative cafeteria Phase II (study days 49 – 92)

		**Ratio of calories from protein / total calories**		
**Group A**	**Group B**	*Difference*	*95% CI*	*P adj*
**RYGB+OM+L**	**RYGB+OM**	-0.00368	-0.005- -0.002	<0.001
**Sham+OM**	**RYGB+OM**	-0.03181	-0.034 - -0.03	<0.001
**Sham+OM+L**	**RYGB+OM**	-0.02797	-0.029 - -0.026	<0.001
**Sham+OM**	**RYGB+OM+L**	-0.02812	-0.029 - -0.026	<0.001
**Sham+OM+L**	**RYGB+OM+L**	-0.02429	-0.026 – 0.0225	<0.001
**Sham+OM+L**	**Sham+OM**	0.003834	0.002 – 0.006	<0.001

C. **Postoperative cafeteria Phase I (study days 37 – 48)**

		**Ratio of calories from non-sugar carbohydrate / total calories**		
**Group A**	**Group B**	*Difference*	*95% CI*	*P adj*
**RYGB+OM+L**	**RYGB+OM**	0.051878	0.006 – 0.097	0.019
**Sham+OM**	**RYGB+OM**	-0.27867	-0.32 - -0.233	<0.001
**Sham+OM+L**	**RYGB+OM**	-0.20915	-0.25 - -0.163	<0.001
**Sham+OM**	**RYGB+OM+L**	-0.33055	-0.37 - -0.286	<0.001
**Sham+OM+L**	**RYGB+OM+L**	-0.26102	-0.3 - -0.217	<0.001
**Sham+OM+L**	**Sham+OM**	0.069524	0.025 – 0.11	0.0003

Postoperative cafeteria Phase II (study days 49 – 92)

		**Ratio of calories from non-sugar carbohydrate / total calories**		
**Group A**	**Group B**	*Difference*	*95% CI*	*P adj*
**RYGB+OM+L**	**RYGB+OM**	0.092439	0.067 – 0.118	<0.001
**Sham+OM**	**RYGB+OM**	-0.2319	-0.26 - -0.21	<0.001
**Sham+OM+L**	**RYGB+OM**	-0.17825	-0.2 - -0.15	<0.001
**Sham+OM**	**RYGB+OM+L**	-0.32434	-0.35 - -0.3	<0.001
**Sham+OM+L**	**RYGB+OM+L**	-0.27069	-0.29 - -0.246	<0.001
**Sham+OM+L**	**Sham+OM**	0.053648	0.029 – 0.078	<0.001

D. Postoperative cafeteria Phase I (study days 37 – 48)

		Ratio of calories from sugar carbohydrate / total calories		
**Group A**	**Group B**	*Difference*	*95% CI*	*P adj*
**RYGB+OM+L**	**RYGB+OM**	0.007972	-0.019 – 0.035	0.87
**Sham+OM**	**RYGB+OM**	0.108215	0.081 – 0.135	<0.001
**Sham+OM+L**	**RYGB+OM**	0.066468	0.039 – 0.093	<0.001
**Sham+OM**	**RYGB+OM+L**	0.100243	0.074 – 0.126	<0.001
**Sham+OM+L**	**RYGB+OM+L**	0.058496	0.032 – 0.084	<0.001
**Sham+OM+L**	**Sham+OM**	-0.04175	-0.068 - -0.015	0.0002

Postoperative cafeteria Phase II (study days 49 – 92)

		Ratio of calories from sugar carbohydrate / total calories		
**Group A**	**Group B**	*Difference*	*95% CI*	*P adj*
**RYGB+OM+L**	**RYGB+OM**	-0.12672	-0.148 - -0.11	<0.001
**Sham+OM**	**RYGB+OM**	-0.06789	-0.089 - -0.047	<0.001
**Sham+OM+L**	**RYGB+OM**	-0.08737	-0.11 - -0.066	<0.001
**Sham+OM**	**RYGB+OM+L**	0.058826	0.0386 – 0.079	<0.001
**Sham+OM+L**	**RYGB+OM+L**	0.039351	0.019 – 0.059	<0.001
**Sham+OM+L**	**Sham+OM**	-0.01948	-0.0397 – 0.001	0.064
